# Pomalidomide and Dexamethasone Are Effective in Relapsed or Refractory Multiple Myeloma in a Real-Life Setting: A Multicenter Retrospective Study in Taiwan

**DOI:** 10.3389/fonc.2021.695410

**Published:** 2021-05-28

**Authors:** Yu-Chin Hung, Jyh-Pyng Gau, Shang-Yi Huang, Bor-Sheng Ko, Chieh-Lin Jerry Teng

**Affiliations:** ^1^ Division of Hematology, Department of Internal Medicine, National Taiwan University Hospital Yunlin Branch, Yunlin, Taiwan; ^2^ Division of Hematology and Oncology, Department of Medicine, Taipei Veterans General Hospital, Taipei, Taiwan; ^3^ School of Medicine, National Yang Ming Chiao Tung University, Taipei, Taiwan; ^4^ Division of Hematology, Department of Internal Medicine, National Taiwan University Hospital, Taipei, Taiwan; ^5^ Department of Hematological Oncology, National Taiwan University Cancer Center, Taipei, Taiwan; ^6^ Division of Hematology/Medical Oncology, Department of Medicine, Taichung Veterans General Hospital, Taichung, Taiwan; ^7^ Department of Life Science, Tunghai University, Taichung, Taiwan; ^8^ School of Medicine, Chung Shan Medical University, Taichung, Taiwan

**Keywords:** multiple myeloma, pomalidomide, treatment response, refractory, relapse

## Abstract

**Background:**

The therapeutic options of relapsed or refractory multiple myeloma (RRMM) remain a challenge. The MM-003 trial demonstrated that RRMM patients treated with pomalidomide and dexamethasone (Pom/Dex) have better progression-free survival (PFS) than those treated with high-dose dexamethasone alone. However, the real-world effectiveness of Pom/Dex in these patients in Taiwan remains unclear.

**Methods:**

This multicenter, registry-based study retrospectively reviewed the medical records of 49 consecutive patients undergoing Pom/Dex treatment for RRMM. We investigated the overall response rate (ORR) and PFS in these patients. The patients were stratified into two groups: those who received two (n=33) and those who received more than two (n=16) prior lines of treatment according to the numbers of regimens before Pom/Dex therapy. The differences in ORR and PFS between these two groups were further analyzed. We also analyzed factors attributed to disease progression.

**Results:**

The ORR was 47.7%, and the median PFS was 4.0 months (range, 0.1−21.1). Patients who received two prior lines of treatment had a higher ORR than those who received more than two prior lines of treatment (55.2% *vs.* 33.3%; p=0.045). The median PFS of these groups was 4.8 and 3.9 months, respectively (p=0.805). Primary lenalidomide refractoriness reduced the risk of myeloma progression following Pom/Dex treatment (hazard ratio, 0.14; p=0.001).

**Conclusions:**

The median PFS following Pom/Dex treatment in Taiwanese RRMM patients in a real-world setting was similar to that reported by the MM-003 trial. Primary lenalidomide refractoriness should not be an obstacle for Pom/Dex treatment in RRMM.

## Introduction

Multiple myeloma (MM) is a hematologic malignancy that clinically presents with anemia, bone pain, impaired renal function, and hypercalcemia. Abnormal plasma cell proliferation is the primary pathophysiology of MM (1). Although MM remains an incurable disease, the treatment outcome of MM has significantly improved over the past decade. One reason for such improvement is the induction treatment with bortezomib. In the VISTA trial, transplant-ineligible newly diagnosed MM patients undergoing induction therapy of bortezomib plus melphalan and prednisone were found to have better progression-free survival (PFS) and overall survival (OS) than those treated with melphalan and prednisolone (2). Besides, both bortezomib-thalidomide-dexamethasone and bortezomib-cyclophosphamide-dexamethasone inductions provide a response rate of as high as 80% to transplant eligible newly diagnosed MM patients (3).

Unfortunately, relapse seems to be inevitable in MM. Although therapeutic options for relapsed or refractory MM (RRMM) are significantly increasing, treatment of RRMM remains a critical challenge. Currently, lenalidomide with dexamethasone (4, 5) or lenalidomide-dexamethasone-based regimens (6, 7) are one of the primary treatment options for MM patients who are refractory to or experience relapse from bortezomib-based induction therapies in Taiwan. However, further therapeutic strategies become limited when the disease does not respond to or further relapses following treatment with lenalidomide-dexamethasone-based regimens. Newer generations of proteasome inhibitors or immunomodulatory drugs (IMiDs)-based regimens might facilitate the disease control under this clinical scenario. One of the examples comes from the ENDEAVOR study. Patients with RRMM who underwent carfilzomib and dexamethasone had a longer median PFS than those treated with bortezomib and dexamethasone (18.7 *vs.* 9.4 months; p <0.0001) (8). In terms of the newer generation IMiDs-based treatment, pomalidomide-dexamethasone (Pom/Dex) could be a promising therapeutic option.

Pomalidomide is an analog of thalidomide and lenalidomide. As the latest IMiD, pomalidomide is more potent and less toxic than thalidomide and lenalidomide (9). The precise mechanism of pomalidomide’s antimyeloma effect is not fully understood. The inhibition of nuclear factor κ-B and apoptosis induction *via* the caspase 8/death receptor pathway could be partially responsible for pomalidomide’s direct antimyeloma effects (10). Impeding cytokine production, immunomodulation, and tumor microenvironment interactions could be associated with its indirect antimyeloma activity (11). The primary evidence of pomalidomide’s clinical benefits in RRMM comes from the MM-003 study (12). This randomized, open-labeled, phase 3 trial demonstrated that MM patients who had failed at least two prior bortezomib and lenalidomide treatments had a longer median PFS by Pom/Dex treatment than high-dose dexamethasone alone (4.0 *vs.* 1.9 months, p <0.0001). However, the real-world effectiveness of Pom/Dex in these particular patients is unclear and could differ from that demonstrated in clinical trials.

Therefore, we evaluated the real-world RRMM patients’ outcomes undergoing Pom/Dex therapy. We also compared the overall response rate (ORR) and PFS among patients treated with Pom/Dex as third or more than third-line therapies. Variables attributed to disease progression following Pom/Dex treatment were also studied.

## Materials and Methods

### Ethics

This study was approved by the review board of the institutions participating in this research (Taipei Veterans General Hospital: 2019-04-006AC, National Taiwan University Hospital: 202101021RINA, and Taichung Veterans General Hospital: CE20071B) and has been conducted in accordance with the Declaration of Helsinki. The institutional review board agreed to waive patients’ informed consent because of the retrospective study design.

### Patients

This retrospective study enrolled consecutive RRMM patients of age ≥20 years who were treated with at least two previous regimens and had undergone treatment with the Pom/Dex regimen from February 2016 to March 2020. Patients without a history of regular follow-up and those with non-secretory MM were excluded. Finally, 49 RRMM patients fulfilled these criteria. The patients were stratified into two groups: those who received two (n = 33) and those who received more than two (n = 16) prior lines of treatment according to the numbers of regimens before Pom/Dex therapy.

### Dose Adjustment and Adverse Events

The Pom/Dex regimen in the current study contained daily pomalidomide 4 mg and weekly dexamethasone 20 mg on days 1−21 during each 28-day cycle. Our patients did not routinely receive the weekly dexamethasone 40 mg as designed in the MM-003 study because most Asians have a lower body surface area than Caucasians. We assessed the Pom/Dex regimen’s toxicity using the National Cancer Institute’s Common Terminology Criteria for Adverse Events (CTCAE v4.0). In patients with grade 3−4 adverse events (AEs) attributed to Pom/Dex regimen, the pomalidomide dose or interval was adjusted by a sequential reduction (13).

### Outcome Measures

The Pom/Dex treatment response was evaluated by the International Myeloma Working Group consensus criteria (14). The treatment response was not accessible in five patients because of early mortality or AEs. Because we did not routinely conduct bone marrow examinations to assess the treatment response in our study cohort, stringent complete remission was not a part of the response assessment. The PFS was defined as the period from the date of Pom/Dex therapy initiation to the date of Pom/Dex therapy cessation due to progression or end of the analysis (November 30, 2020).

We also investigated whether previous therapeutic exposure would impact the Pom/Dex treatment outcome. For this purpose, we compared the best response and median PFS between the two treatment groups. We did not compare PFS2 and overall survival between the groups because treatments following the Pom/Dex regimen were considerably diverse (**Supplementary Table 1**).

### Statistical Analyses

We used the Mann-Whitney *U* test to compare the continuous variables and the chi-squared test to compare categorical variables between the two groups. The PFS with Pom/Dex treatment was analyzed using the log-rank test. As quantified by hazard ratios (HRs) and accompanying 95% confidence intervals (CIs), we used Cox proportional hazards regression to investigate the variables which were attributed to the disease progression following the Pom/Dex treatment. A p-value of <0.05 was considered statistically significant. SPSS version 20.0 (SPSS Inc., Chicago, IL, USA) was used to perform all statistical analyses.

## Results

### Patient Demographics

The average age of the 49 patients in this study was 65.6 (range, 29−87) years. Using the Durie-Salmon staging (DSS) system, 71.4% (35/49) of the patients were found to be at stage III MM during the initial diagnosis. Besides, 44.9% (22/49) of the patients were found to have stage III MM according to the international staging system (ISS). Among the 49 patients, only one did not receive any bortezomib-containing regimen before Pom/Dex treatment. All 49 RRMM patients had exposure to lenalidomide-based treatments upon initiation of Pom/Dex therapy. Among the 49 patients, 47 patients stopped lenalidomide because of disease refractoriness or progression, and two discontinued lenalidomide due to AEs. With respect to autologous hematopoietic stem cell transplantation (auto-HSCT), 42.9% (21/49) of the patients had undergone auto-HSCT.

With regard to the clinical characteristics of the two groups of RRMM patients, both groups had a comparable sex distribution (p = 0.105), stages at diagnosis (p = 0.366 for ISS; p = 0.725 for DSS), disease subtypes (p = 0.653), primary bortezomib refractoriness (p = 1.000), and renal function impairment (p = 0.386). However, patients who received two prior lines of treatment were younger (63.0 *vs.* 71.0 years; p = 0.007) and had a higher percentage of primary lenalidomide refractoriness (42.4% *vs.* 0%; p = 0.002) than those who received more than two prior lines of treatment (**Table 1**).

**Table 1:** Clinical characteristics of patients undergoing Pom/Dex treatment.

Parameters	All patients(n = 49)		Two prior lines of treatment (n = 33)		More than two prior lines of treatment (n = 16)			p-value
**Age (years)**	65.6 ± 10.7		63.0 ± 11.4			71.0 ± 6.5		0.007^a^
**Sex, n (%)**								
Female	25	(51.0)	20	(60.6)		5	(31.3)	0.105^b^
Male	24	(49.0)	13	(39.4)		11	(68.8)	
**ISS, n (%)**								
I	10	(20.4)	8	(24.2)		2	(12.5)	0.366^b^
II	14	(28.6)	11	(33.3)		3	(18.8)	
III	22	(44.9)	12	(36.4)		10	(62.5)	
Missing	3	(6.1)	2	(6.1)		1	(6.3)	
**DSS, n (%)**								
I	5	(10.2)	4	(12.1)		1	(7.1)	0.725^b^
II	5	(10.2)	4	(12.1)		1	(7.1)	
III	35	(71.4)	23	(69.7)		12	(85.7)	
Missing	4	(8.2)	2	(6.1)		2	(12.5)	
**Disease subtypes, n (%)**								
IgG	23	(46.9)	15	(45.5)		8	(50.0)	0.653^b^
IgA	10	(20.4)	8	(24.2)		2	(12.5)	
Light chain disease	15	(30.6)	9	(27.3)		6	(37.5)	
Others	1	(2.0)	1	(3.0)		0	(0.0)	
**Primary bortezomib refractoriness, n (%)**								
Yes	1	(2.0)	1	(3.0)		0	(0.0)	1.000^b^
No	48	(98.0)	32	(97.0)		16	(100.0)	
**Primary lenalidomide refractoriness, n (%)**								
Yes	14	(28.6)	14	(42.4)		0	(0.0)	0.002^b^
No	35	(71.4)	19	(57.6)		16	(100.0)	
**Auto-HSCT, n (%)**								
Yes	21	(42.9)	17	(51.5)		4	(25.0)	0.147^b^
No	28	(57.1)	16	(48.5)		12	(75.0)	
**Creatinine >2 mg/dL, n (%)**								0.386^b^
Yes	6	(12.5)	3	(9.4)		3	(18.8)	
No	42	(87.5)	29	(90.6)		13	(81.3)	
**Leukocyte (10^3^/μL)**	4.1 ± 1.8		3.8 ± 1.6			4.6 ± 2.1		0.214^a^
**Hemoglobin (g/dL)**	9.8 ± 2.3		9.7 ± 2.6			9.9 ± 1.5		0.645^a^
**Platelet (10^3^/μL)**	125.3 ± 80.0		122.8 ± 87.1			130.1 ± 66.4		0.466^a^

The numerical data are presented as the mean ± standard error of the mean.

Pom/Dex, pomalidomide and dexamethasone; ISS, international staging system; DSS, Durie–Salmon staging system; IgG, immunoglobulin G; IgA, immunoglobulin A; auto-HSCT, autologous hematopoietic stem cell transplantation.

^a^The data were compared using the Mann-Whitney U test.

^b^The data were compared using the chi-squared test.

### Treatment Response and PFS

The ORR of Pom/Dex treatment to RRMM was 47.7% (21/44). The median Pom/Dex treatment duration was 4.0 months (range, 0.1−21.1). Disease progression was the primary reason for Pom/Dex discontinuation (40/49; 81.6%). Besides, eight of the 49 patients (12.3%) stopped Pom/Dex treatment because of AEs. Only one patient was continuing Pom/Dex treatment on the day when this study was censored. **Table 2** enumerates these results.

**Table 2:** Outcome comparison among patients who received two and more than two prior therapies.

Parameters	All patients(n = 49)		Two prior lines of treatment (n = 33)		More than two prior lines of treatment (n = 16)		p-value
**Best response by Pom/Dex, n (%)**							
CR	1	(2.0)	1	(3.0)	0	(0.0)	0.045^a^
VGPR	5	(10.2)	4	(12.1)	1	(6.3)	
PR	15	(30.6)	11	(33.3)	4	(25.0)	
SD	16	(32.7)	6	(18.2)	10	(62.5)	
Refractory disease	7	(14.3)	7	(21.2)	0	(0.0)	
Non-accessible	5	(10.2)	4	(12.1)	1	(6.3)	
**Reasons for Pom/Dex discontinuation (n = 48)**							
Progression	40	(83.3)	26	(81.3)	14	(87.5)	0.701^a^
Adverse events	8	(16.7)	6	(18.8)	2	(12.5)	
**Median duration of Pom/Dex treatment, months (median, range)**	4.0	0.1−21.1	2.9	0.1−21.1	4.7	1.2−14.1	0.254^b^

Pom/Dex, pomalidomide and dexamethasone; CR, complete remission; VGPR, very good partial response; PR, partial response; SD, stable disease.

^a^The data were compared using the Mann-Whitney U test.

^b^The data were compared using the chi-squared test.

The response rates in patients who received two and those who received more than two prior lines of treatment were 55.2% (16/29) and 33.3% (5/15), respectively. Patients exposed to two prior lines of regimens before the Pom/Dex had a higher ORR than those exposed to more than two prior lines of treatment (p = 0.045). Notably, these two groups had a similar treatment duration (p = 0.254) and causes of Pom/Dex discontinuation (p = 0.701). Disease progression remained the leading cause of Pom/Dex cessation for each group. None of the patients discontinued Pom/Dex due to drug unavailability.

**Figure 1:**
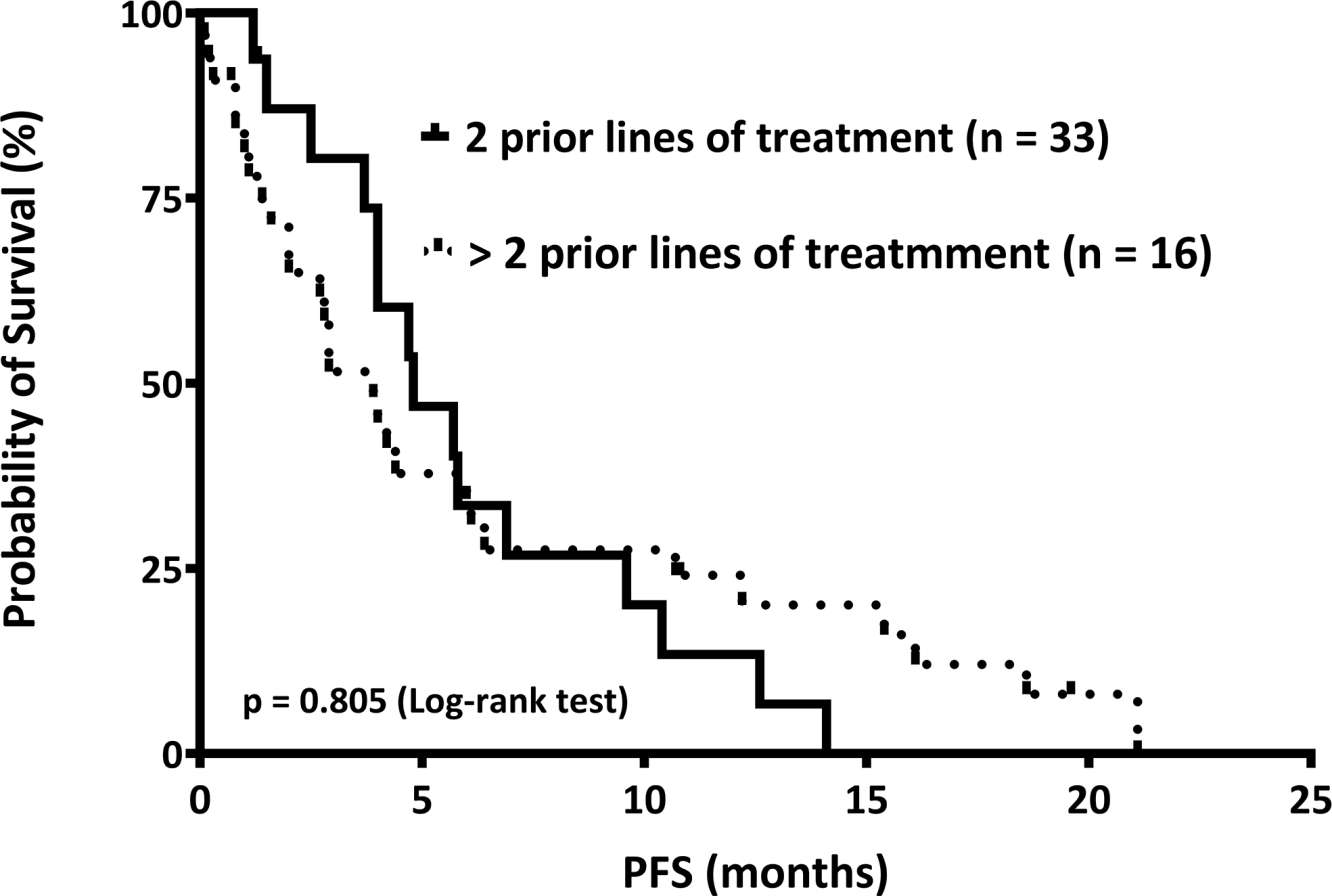
The median progression-free survival (PFS) is shown. We stratified relapsed or refractory multiple myeloma patients into two groups: those who received two (n = 33) and those who received more than two (n = 16) prior lines of treatment, according to the numbers of regimens before pomalidomide and dexamethasone therapy. The PFS of these two groups was 4.8 and 3.9 months, respectively (p = 0.805).

Furthermore, the median PFS in patients who received two and those who received more than two prior lines of treatment were 4.8 and 3.9 months, respectively. Patients with RRMM undergoing Pom/Dex after two prior lines of treatment had a numerically longer PFS than those who received more than two prior lines of treatment; however, the difference was not statistically significant (p = 0.805) (**Figure 1**).

### Variables Attributed to Disease Progression Following Pom/Dex Treatment

We investigated the potential factors attributed to disease progression following Pom/Dex treatment. Because only one patient in our study was primarily refractory to bortezomib, the association between primary bortezomib refractoriness and disease progression was not analyzed. Briefly, the univariate analysis showed that age [hazard ratio (HR), 1.02; 95% CI, 0.99−1.05; p = 0.249], sex (HR, 1.01; 95% CI, 0.55−1.85; p = 0.978), the Eastern Cooperative Oncology Group (ECOG) PS (performance status) of >2 (HR, 1.76; 95% CI, 0.68−4.56; p = 0.247), and auto-HSCT (HR, 0.87; 95% CI, 0.47−1.61; p = 0.650) were not associated with disease progression following the Pom/Dex treatment. Importantly, primary lenalidomide refractoriness was significantly associated with less myeloma progression (HR, 0.42; 95% CI, 0.18−0.98; p = 0.046).

The multivariate analysis validated the results in which age (HR, 1.04; 95% CI, 1.00−1.09; p = 0.0.059), sex (HR, 1.25; 95% CI, 0.63−2.48; p = 0.523), and auto-HSCT (HR, 1.24; 95% CI, 0.57−2.70; p = 0.592) were not associated with myeloma progression. Primary lenalidomide refractoriness remained a substantial factor attributed to less disease progression following the Pom/Dex (HR, 0.14; 95% CI, 0.04−0.47; p = 0.001). Of note, ECOG PS of >2 significantly increased the risk of disease progression among myeloma patients undergoing Pom/Dex in the multivariate analysis (HR, 5.56; 95% CI, 1.48−20.91; p = 0.011).

Importantly, both the univariate (HR, 1.08; 95% CI, 0.56−2.07; p = 0.814) and multivariate (HR, 0.46; 95% CI, 0.20−1.06; p = 0.068) analyses failed to demonstrate that Pom/Dex was more effective in RRMM patients who received two prior lines of treatment than in those who received more than two prior lines of treatment (**Table 3**).

**Table 3:** Factors associated with progression following Pom/Dex.

Parameters	Univariate analysis			Multivariate analysis		
	HR	95% CI	p*-*value	HR	95% CI	p-value
Age	1.02	0.99−1.05	0.249	1.04	1.00−1.09	0.059
Sex (male *vs.* female)	1.01	0.55−1.85	0.978	1.25	0.63−2.48	0.523
ECOG PS (>2 *vs.* ≤ 2)	1.76	0.68−4.56	0.247	5.56	1.48−20.91	0.011
More than two prior lines of treatment *vs.* two prior lines of treatment	1.08	0.56−2.07	0.814	0.46	0.20−1.06	0.068
Auto-HSCT (yes *vs.* no)	0.87	0.47−1.61	0.650	1.24	0.57−2.70	0.592
Primary lenalidomide refractoriness (yes *vs.* no)	0.42	0.18−0.98	0.046	0.14	0.04−0.47	0.001

Pom/Dex, pomalidomide and dexamethasone; ECOG PS, Eastern Cooperative Oncology Group performance status; auto-HSCT, autologous hematopoietic stem cell transplantation; HR, hazard ratio; CI, confidence interval.

### Adverse Events

With regards to hematologic AEs, leukopenia was the most common, accounting for 8.2% (4/49) of the patients. In addition, skin rash was the most common non-hematologic AE (6.1%, 3/49). Notably, the incidences of various hematologic and non-hematologic AEs were not significantly different between the two treatment groups (**Table 4**). Overall, 16.3% (8/49) of the patients withdrew Pom/Dex treatment due to AEs.

**Table 4:** Adverse events.

Parameters	All patients (n = 49)				Two prior lines of treatment (n = 33)				More than two prior lines of treatment (n = 16)				p-value^*^
	All grades		≥ Grade 3		All grades		≥ Grade 3		All grades		≥ Grade 3		
	n	(%)	n	(%)	n	(%)	n	(%)	n	(%)	n	(%)	
**Hematologic adverse events**													
Anemia	2	4.1	1	2.0	1	3.0	1	3.0	1	6.3	0	0.0	1.000
Leukopenia	4	8.2	3	6.1	3	9.1	3	9.1	1	6.3	0	0.0	0.541
Thrombocytopenia	2	4.1	2	4.1	1	3.0	1	3.0	1	6.3	1	6.3	1.000
**Non-hematologic events**													
Skin rash	3	6.1	2	4.1	1	3.0	1	3.0	2	12.5	1	6.3	1.000
Fatigue	0	0.0	0	0.0	0	0.0	0	0.0	0	0.0	0	0.0	
Peripheral neuropathy	0	0.0	0	0.0	0	0.0	0	0.0	0	0.0	0	0.0	
Edema	0	0.0	0	0.0	0	0.0	0	0.0	0	0.0	0	0.0	
Bacteremia	1	2.0	1	2.0	1	3.0	1	3.0	0	0.0	0	0.0	1.000
Elevated ALT	1	2.0	0	0.0	1	3.0	0	0.0	0	0.0	0	0.0	
GI bleeding	1	2.0	1	2.0	0	0.0	0	0.0	1	6.3	1	6.3	0.327

ALT, alanine aminotransferase; GI, gastrointestinal.

^*^Comparison of ≥ Grade 3 adverse events among patients who received two and those who received more than two prior therapies.

## Discussion

The current study demonstrated that the ORR of Pom/Dex treatment in RRMM patients who had been exposed to at least two prior lines of treatment in Taiwan was 47.7%. Moreover, the median PFS in these patients was 4.0 months (range, 0.1−21.1). Patients who received two prior lines of treatment had a higher ORR than those who received more than two prior lines of treatment (55.2 *vs.* 33.3%; p = 0.045). Furthermore, patients who received two prior lines of treatment had a numerically superior median PFS than those who received more than two prior lines of treatment (4.8 *vs.* 3.9 months). However, the difference was not statistically significant (p = 0.805). Primary lenalidomide refractoriness reduced the risk of disease progression (p = 0.046 by univariate analysis; p = 0.001 by multivariate analysis). Disease progression remained the most common reason for Pom/Dex withdrawal, accounting for 81.6% of the patients.

The real-world data on RRMM patients treated with Pom/Dex might differ from that of phase 3 randomized-control studies. Additionally, real-world data from different study groups could be considerably heterogeneous. A retrospective study from a Polish group showed that, with an ORR of 39.1%, the median PFS of RRMM patients treated with the Pom/Dex regimen could be as high as 10 months (15). However, an Australian group showed that the median PFS in RRMM patients treated with Pom/Dex was only 3.4 months (16). Various patient enrollment criteria, patient monitoring schedules, and different supportive care resources in each institution could be the major reasons for this clinical diversity (**Table 5**).

**Table 5:** Clinical studies of Pom/Dex in relapsed or refractory multiple myeloma.

	Study design	Number of patients	Main findings
Miguel et al. (12)	Randomized, open labeled, phase 3 study	Pom/Dex (n=302) High dose dexamethasone (n=153)	ORR with Pom/Dex was 31% *vs.* 10% with high-dose dexamethasone (p<0·0001) Median PFS with Pom/Dex was 4·0 months *vs.* 1·9 months with high-dose dexamethasone (HR: 0·48; 95% CI: 0·39–0·60; p<0·0001)
Charlinski et al. (15)	Multicenter, retrospective, observational study	Pom/Dex (n=50)	ORR: 39.1% Median PFS: 10.0 months Previous treatments with immunomodulatory drugs, bortezomib or stem cell transplant had no impact on PFS
Scott et al. (16)	Multicenter, retrospective, observational study	Pom/Dex (n=87)	ORR: 32% Median PFS: 3.4 months Patients < 65 years had inferior ORR compared to those aged ≥ 65 years (23% *vs.* 44%, p=0.006)
Current study	Multicenter, retrospective, observational study	Pom/Dex (n=49)	ORR: 47.7%, Median PFS: 4.0 months Primary lenalidomide refractoriness reduced the risk of myeloma progression (HR: 0.14; 95% CI: 0·04–0·47; p=0.001)

Pom/Dex, Pomalidomide and dexamethasone; ORR, overall response rate; PFS, progression free survival; HR, hazard ratio; CI, confidence interval.

Notably, the outcome of RRMM patients treated with Pom/Dex in our study was quite comparable to that of the MM-003 trial. The MM-003 study revealed an ORR of 31% and a median PFS of 4.0 months in RRMM patients treated with the Pom/Dex regimen (12). We found an ORR of 47.7% and a median PFS of 4.0 months (**Table 5**). One of the reasons for the similarity between the results of the MM-003 trial and those of our study is the comparable patient characteristics. All patients in MM-003 were exposed to both bortezomib and lenalidomide. In our study, only a single patient did not receive bortezomib before Pom/Dex treatment. Furthermore, all patients in our cohort were treated with lenalidomide-based regimens until disease progression or intolerable AEs, which was similar to the MM-003 study design. A higher proportion of patients in the MM-003 study underwent auto-HSCT than in our study (71% *vs.* 43%). However, auto-HSCT was not significantly associated with disease progression following the Pom/Dex treatment in our analysis.

Our data showed that patients with less prior treatment exposure were more responsive to Pom/Dex. In contrast, this result was not fully supported by an Italian study. Mele et al. (17) demonstrated that RRMM patients who received two, three, or more than three previous lines of therapy had a similar ORR following Pom/Dex treatment (31% *vs.* 31% *vs.* 38%, respectively). Nevertheless, our patients who received two prior lines of therapy did not have a significantly longer PFS than those who received more than two prior lines of treatment. This result suggests that a higher response rate might not necessarily translate into a better PFS in heavily treated RRMM.

In the current study, primary lenalidomide refractoriness was a substantial factor associated with less disease progression following Pom/Dex treatment. This suggests that the primary refractoriness of one particular IMiD does not always result in a worse response to another IMiD. The precise reason behind this clinical observation remains unclear. Although thalidomide, lenalidomide, and pomalidomide share a similar chemical structure, their anti-myeloma mechanisms differ substantially (18). Furthermore, less IMiD exposure possibly induces fewer clonal evaluations of myeloma cells, which makes the subsequent IMiD more effective. Notably, this result could also presents with a statistical bias due to the small number of patients in the current study. More studies are needed to understand the underlying mechanism of this phenomenon.

In terms of Pom/Dex-associated adverse effects, there were fewer reported AEs in our study than those reported in the MM-003 study. A retrospective study design, less intensive patient surveillance, and a more adjusted dosing schedule could be the primary reasons for this discrepancy. Nevertheless, only 16.3% of our study patients had Pom/Dex treatment withdrawn due to intolerable adverse effects, suggesting Pom/Dex remained a tolerable regimen to RRMM in a real-life scenario in Taiwan.

The small sample size and the retrospective study design were the major limitations of this study. Besides, our study could not analyze the impact of cytogenetics on Pom/Dex efficacy because we did not routinely obtain bone marrow tissues before Pom/Dex initiation. Furthermore, the treatment response to Pom/Dex was not accessible in five patients because of their poor general conditions or rapid development of treatment-associated AEs.

In conclusion, our study demonstrated that the median PFS of Pom/Dex in RRMM patients was 4.0 months in a real-world setting in Taiwan. Less previous treatment exposure might enhance the treatment response, but not PFS, in RRMM patients undergoing Pom/Dex therapy. Prospective studies with larger cohorts and randomized study designs are required to validate our results in the future. Primary lenalidomide refractoriness should not be an obstacle to Pom/Dex treatment in RRMM patients. Adding elotuzumab (19) or isatuximab (20) to Pom/Dex could be a solution to improve the efficacy of this regimen in future practice.

## Data Availability Statement

The original contributions presented in the study are included in the article/**Supplementary Material**. Further inquiries can be directed to the corresponding author.

## Ethics Statement

The studies involving human participants were reviewed and approved by Taipei Veterans General Hospital: 2019-04-006AC, National Taiwan University Hospital: 202101021RINA, and Taichung Veterans General Hospital: CE20071B. Written informed consent for participation was not required for this study in accordance with the national legislation and the institutional requirements.

## Author Contributions

Y-CH performed the study and analyzed the data. J-PG analyzed the data and critically reviewed the manuscript. S-YH performed the research and critically reviewed the manuscript. B-SK designed the study and critically reviewed the manuscript. C-LT designed the study, interpreted the data, and wrote the manuscript. All authors contributed to the article and approved the submitted version.

## Funding

This study was supported by grants from the Multiple Myeloma Working Group, the Hematology Society of Taiwan.

## Conflict of Interest

C-LT received an honorarium and consulting fees from Novartis, Roche, Takeda, Johnson & Johnson, Amgen, BMS Celgene, Kirin, AbbVie, and MSD.

The remaining authors declare that the research was conducted in the absence of any commercial or financial relationships that could be construed as a potential conflict of interest.
