# Supplementary material for: Pomalidomide and Dexamethasone Are Effective in Relapsed or Refractory Multiple Myeloma in a Real-Life Setting: A Multicenter Retrospective Study in Taiwan
Source: Front Oncol. 2021 May 28;11:695410. doi: 10.3389/fonc.2021.695410 (PMC8193980; doi:10.3389/fonc.2021.695410)
Supplement: Supplementary file 1 [file Table_1.doc]

| **Supplemental table 1. Treatments after Pom/Dex (n = 48)** | | |
| --- | --- | --- |
| **Treatments** | **n** | **(%)** |
| Supportive care | 16 | 33.3 |
| Chemotherapy | 10 | 20.8 |
| Carfilzomib-based regimens | 10 | 20.8 |
| Previously used regimens | 5 | 10.5 |
| Daratumumab-based regimens | 4 | 8.3 |
| Loss follow-up | 2 | 4.2 |
| Clinical trial | 1 | 2.1 |
| Pom/Dex: pomalidomide with dexamethasone  ^*^One patient remained with Pom/Dex | | |
